# Ethnic disparity in pneumonia-specific mortality among children under 5 years of age in Sichuan Province of Western China from 2010 to 2017

**DOI:** 10.1186/s12889-019-8056-7

**Published:** 2019-12-23

**Authors:** Min Luo, Ziling Zhao, Linkun He, Bingzhong Su, Weixin Liu, Gang Zhang

**Affiliations:** Sichuan Provincial Maternal and Child Health Hospital, No.290, West Shayan Second Street, Chengdu, Sichuan 610045 People’s Republic of China

**Keywords:** Pneumonia, Ethnic disparity, Children under five, Mortality

## Abstract

**Background:**

To reveal the ethnic disparity in the pneumonia-specific mortality rates of children under the age of 5 years (PU5MRs) and provide suggestions regarding priority interventions to reduce preventable under-five-years-of-age deaths.

**Methods:**

Data were obtained from the Direct Report System of Maternal and Child Health in Sichuan. The Cochran-Armitage trend test was used to assess the time trend. The Cochran-Mantel-Haenszel test and Chi-square test were used to examine the differences in the PU5MRs among different groups.

**Results:**

The PU5MRs in the minority and nonminority counties decreased by 53.7 and 42.3% from 2010 to 2017, respectively. The PU5MRs of the minority counties were 4.81 times higher than those of the nonminority counties in 2017. The proportion of pneumonia deaths to total deaths in Sichuan Province increased from 11.7% in 2010 to 15.5% in 2017. The pneumonia-specific mortality rates of children in the categories of 0–28 days, 29 days-11 months, and 12–59 months were reduced by 55.1, 38.8, and 65.5%, respectively, in the minority counties and by 35.5, 43.1, and 43.7%, respectively, in the nonminority counties.

**Conclusions:**

PU5MRs declined in Sichuan, especially in the minority counties, while ethnic disparity still exists. Although the PU5MRs decreased more for the minority counties as a fraction of all mortality, the absolute number of such deaths were higher, and therefore more children in these counties continue to die from pneumonia than from the non-minority counties. Priority should be given to strategies for preventing and controlling child pneumonia, especially for postneonates, in the minority counties.

## Background

Pneumonia was once regarded as “the forgotten killer in children” [1], however by 2015 was recognised as the second leading cause of death among children under the age of 5, and it was estimated that approximately 0.920 million children died of pneumonia, accounting for 15.5% of the total deaths of children under the age of five years [2]. The proportion of deaths caused by pneumonia in children under the age of five years decreased from 1996 to 2015 [3]. However, the number of pneumonia deaths among children under the age of 5 years is still large due to the large number of under-5 death [4].

Malnutrition, poverty and inadequate access to health care are important factors influencing the pneumonia-specific mortality rate of children [5]. Approximately 75% of the burden of childhood pneumonia occurs in children from the low- and middle-income areas, where poverty and ambient air pollution prevail [6]. In 2015, the PU5MRs in Central and Eastern Europe and the Commonwealth of Independent States (CEE/CIS) was 2.0‰, whereas it was 13.7‰ in Sub-Saharan Africa [7]. In addition, geographical differences in pneumonia deaths exist not only between different countries but also in different regions of a country [8]. This regional disparity is more pronounced between underdeveloped remote rural areas in western regions and developed rural areas in coastal regions [9, 10]. The proportion of pneumonia deaths among children under 5 years old is higher in Western China than it is in the Eastern and Central regions [3].

Western China is known for its ethnic diversity, with 71% of China’s ethnic minority population and 46 different ethnic groups living there [11, 12]. Sichuan is the most populous province in Western China, and its minority regions account for 62.9% of the total population [13]. According to the 2010 census, the ethnic minority population of Sichuan Province equaled 4.98 million, accounting for 6.1% of the whole population [13]. The sparse population, lack of transportation, underdeveloped economy, unique religious beliefs and living habits have always been important factors restricting the development of the health of local residents in the minority counties [14, 15].Under-five-years-of-age deaths in minority counties account for approximately one-fifth of all deaths, and pneumonia is the leading cause of death among children under 5 years old in Sichuan Province [16].

This study focuses on the ethnic differences in pneumonia death among children under 5 years old in Sichuan Province of Western China, with the aim of providing priority intervention recommendations for the health administration to eliminate preventable deaths from pneumonia in children under 5 years old and sharing the results with other countries that are in similar situations.

## Methods

### Data sources

Sichuan Province consists of 67 minority counties and 116 nonminority counties [17]. Data for this study were obtained from the population-based Direct Report System of Maternal and Child Health (DRSMCH) in Sichuan. In accordance with the unified standard of the National Maternal and Child Health Surveillance System, a level-by-level reporting, review and quality-control network(i.e., village, township/community, county/district, municipality, province) has been established in Sichuan Province. The under-five-years-of-age death data are reported after the information is confirmed, while the live births data are reported yearly. Details of the verification of the causes of death and quality control procedures have been described in previous reports [3, 8, 18].

### Data analysis

The PU5MRwas calculated as the number of children under the age of 5 years who died of pneumonia per 100,000 live births and was adjusted by the annual provincial quality control underreporting rate. The mortality rate in the minority and nonminority counties was calculated in three age groups (i.e., 0–28 days, 29 days-11 months, 12–59 months).

The Cochran-Armitage trend test was used to test the time trends of the pneumonia mortality rate, proportion of pneumonia deaths to total deaths, and proportion of children seeking treatment at any medical institution before death in each region [19]. The Cochran-Mantel-Haenszel (CMH) test was used to calculate relative risks (RRs) and 95%CIs and to compare the risk of pneumonia death in different ethnic regions and age groups [20]. A Chi-square test was used to compare the proportion of pneumonia deaths to total deaths between the minority and nonminority counties. *P* < 0.05 was considered statistically significant.

## Results

### Ethnic disparity in PU5MR

The PU5MR in Sichuan Province dropped by 40.2% (from 197 per 100,000 live births in 2010 to 118 per 100,000 live births in 2017, *χ*^2^ = 106, *p*_*trend*_ < 0.001). The rate decreased by 53.7%in the minority (from 701 to 324 per 100,000 live births, *χ*^2^ = 243, *p*_*trend*_ < 0.001) and 42.3% in the nonminority (from 117 to 67.4 per 100,000 live births, *χ*^2^ = 48.3, *p*_*trend*_ < 0.001) counties. Compared with the nonminority counties, the RR of PU5MR in the minority counties was 5.99 (95% CI: 5.24–6.85) in 2010 and decreased to 4.81 (95% CI: 4.16–5.56) in 2017. With the decrease of PU5MR, the pneumonia deaths as a percent age of total deaths increased in Sichuan Province (from 11.7 to 15.5%, *χ*^2^ = 26.8, *p*_*trend*_ < 0.001) and the nonminority counties (from 7.77 to 10.0%, *χ*^2^ = 13.7, *p*_*trend*_ < 0.001), but the time trend was not observed in the minority counties (*χ*^2^ = 0.591, *p*_*trend*_ = 0.442). During the study period, the proportion of pneumonia deaths to total deaths in the minority counties was higher than that in the nonminority counties every year (all *p* < 0.001) (Table 1).

**Table 1:** The PU5MR and proportion of pneumonia deaths to total deaths by ethnic groups

Time period	Minority counties		Nonminority counties		Total		RR (95% CI) M to Non	Comparison of proportion (χ^2^)
	mortality rate	pneumonia (%)	mortality rate	pneumonia (%)	mortality rate	pneumonia (%)		
2010	700.68	24.40	116.90	7.77	197.23	11.65	5.99[5.24,6.85]	352.01^*^
2011	993.99	35.18	155.28	10.64	275.77	16.64	6.40[5.70,7.18]	556.83^*^
2012	796.20	37.79	135.29	11.27	228.27	17.19	5.89[5.24,6.61]	562.46^*^
2013	694.47	36.74	99.69	9.43	185.23	15.73	6.97[6.17,7.87]	656.23^*^
2014	403.25	30.77	105.22	12.18	165.97	17.38	3.83[3.38,4.35]	264.38^*^
2015	386.99	29.63	97.22	12.37	156.11	17.51	3.98[3.50,4.53]	226.79^*^
2016	366.21	31.02	71.97	9.78	132.12	15.98	5.09[4.41,5.87]	332.67^*^
2017	324.22	28.93	67.41	10.00	117.90	15.47	4.81[4.16,5.56]	268.82^*^

^*^p<0.001

**Fig. 1:**
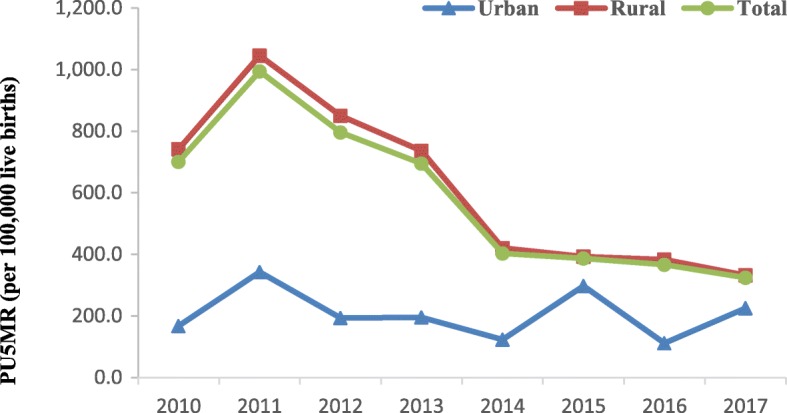
PU5MR in urban and rural areas in minority counties from 2010 to 2017

Furthermore, significant disparities were observed within ethnic minority areas, with consistently higher PU5MRs in rural than in urban areas. Compared with urban areas, the RRs of PU5MRs decreased from 4.44 (95% CI 2.10–9.36) in 2010 to 1.47 (95% CI 1.11–2.33) in 2017 in rural areas (Fig. 1).

### Ethnic disparity in pneumonia-specific mortality rate at different ages

The pneumonia-specific mortality rate of children at 0–28 days, 29 days-11 months and 12–59 months decreased by 55.1% (*χ*^2^ = 766, *p*_*trend*_ < 0.001), 38.8% (*χ*^2^ = 50.4, *p*_*trend*_ < 0.001), and 65.5% (*χ*^2^ = 147, *p*_*trend*_ < 0.001), respectively, in the minority counties and 35.49% (*χ*^2^ = 9.64, *p*_*trend*_ = 0.002), 43.1% (*χ*^2^ = 26.4, *p*_*trend*_ < 0.001), and 43.7% (*χ*^2^ = 14.8, *p*_*trend*_ < 0.001), respectively, in the nonminority counties. The most significant decrease in pneumonia-specific mortality in the minority and nonminority areas was found in the 12–59-month-oldage group. Among the comparisons of different age groups between the minority and nonminority areas, the largest disparities existed in the 12–59 month old age group. There were more differences between different age groups within the minority and nonminority areas, with the highest pneumonia-specific mortality rate in the 29 days-11 month old age group in the minority counties and the lowest pneumonia-specific mortality rate in the 12–59 month old age group in the nonminority counties. Compared with children at 0–28 days, the RRs of pneumonia-specific mortality rates increased from 1.90 (95% CI: 1.48–2.45) to 2.54 (95% CI: 1.97–3.28) in the 29 days-11 month old age group in the minority counties and decreased from 0.540 (95% CI: 0.416–0.701) to 0.485 (95% CI: 0.362–0.651) in the 12–59 month old age group in the nonminority counties (Table 2).

**Table 2:** The pneumonia-specific mortality rates in minority and nonminority by age groups^a^

Areas	Time period	0–28 days(N)	29 days-11 months (PN)	12–59 months(C)	RR (95% CI)				
					M to Non(N)	M to Non (PN)	M to Non(C)	PN to N	C to N
Minority counties(M)	2010	151.99	292.67	257.08	3.54[2.74,4.58]	5.77[4.69,7.09]	11.02[8.47,14.34]	1.90[1.48,2.45]	1.68[1.30,2.18]
	2011	219.54	468.29	325.21	3.61[2.89,4.49]	7.26[6.09,8.66]	9.69[7.72,12.16]	2.16[1.75,2.67]	1.54[1.23,1.92]
	2012	258.20	371.49	245.76	3.91[3.17,4.82]	5.93[4.97,7.09]	10.04[7.87,12.81]	1.66[1.35,2.04]	1.19[0.95,1.48]
	2013	215.69	374.20	206.97	4.12[3.29,5.16]	8.03[6.67,9.66]	10.29[8.01,13.23]	2.06[1.67,2.54]	1.30[1.03,1.63]
	2014	130.54	281.07	92.00	2.03[1.60,2.59]	5.63[4.66,6.81]	4.06[3.10,5.32]	2.59[2.06,3.26]	1.06[0.81,1.39]
	2015	98.72	278.61	93.41	2.24[1.73,2.89]	4.93[4.11,5.92]	4.99[3.77,6.60]	2.79[2.20,3.53]	1.20[0.92,1.59]
	2016	69.09	216.09	97.04	2.00[1.53,2.61]	7.63[6.13,9.48]	8.29[6.00,11.45]	2.99[2.32,3.85]	1.44[1.08,1.91]
	2017	68.20	179.23	88.57	2.46[1.87,3.24]	6.20[5.00,7.70]	6.75[4.97,9.16]	2.54[1.97,3.28]	1.33[1.00,1.77]
Nonminority counties (Non)	2010	42.91	50.75	23.33	–	–	–	1.17[0.95,1.44]	0.54[0.42,0.70]
	2011	60.90	64.51	33.57	–	–	–	1.07[0.89,1.29]	0.57[0.46,0.72]
	2012	66.05	62.60	24.47	–	–	–	1.09[0.912,1.31]	0.46[0.37,0.58]
	2013	52.36	46.60	20.11	–	–	–	1.05[0.86,1.29]	0.52[0.41,0.66]
	2014	64.19	49.90	22.67	–	–	–	0.93[0.76,1.14]	0.53[0.42,0.67]
	2015	44.14	56.52	18.72	–	–	–	1.26[1.03,1.55]	0.54[0.42,0.70]
	2016	34.61	28.34	11.71	–	–	–	0.78[0.62,0.99]	0.35[0.25,0.47]
	2017	27.68	28.89	13.12	–	–	–	1.01[0.80,1.28]	0.48[0.36,0.65]

^a^M, minority counties; Non, nonminority counties; N, PN, 29 days-11 months; C, 12–59 months

**Fig. 2:**
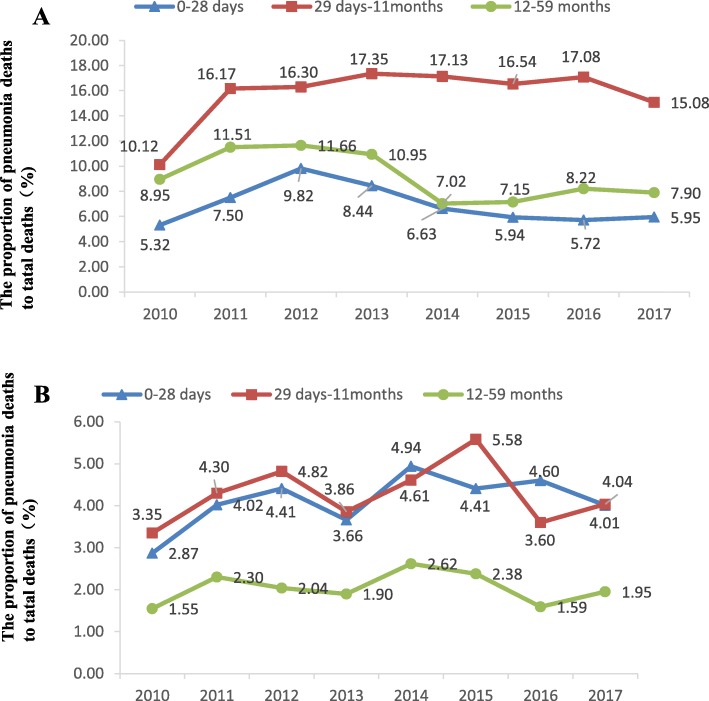
Proportion of pneumonia deaths to total deaths in minority (**a**) and nonminority (**b**) counties

From 2010 to 2017, the proportion of pneumonia deaths to total deaths in the minority counties of all age groups was higher than that in the nonminority counties (all *p* < 0.05). The proportion of pneumonia deaths to total deaths in the minority counties increased in postneonates (*χ*^2^ = 13.3, *p* < 0.001) and decreased in the childhood group (*χ*^2^ = 14.8, *p* < 0.001), respectively (Fig. 2).

**Fig. 3:**
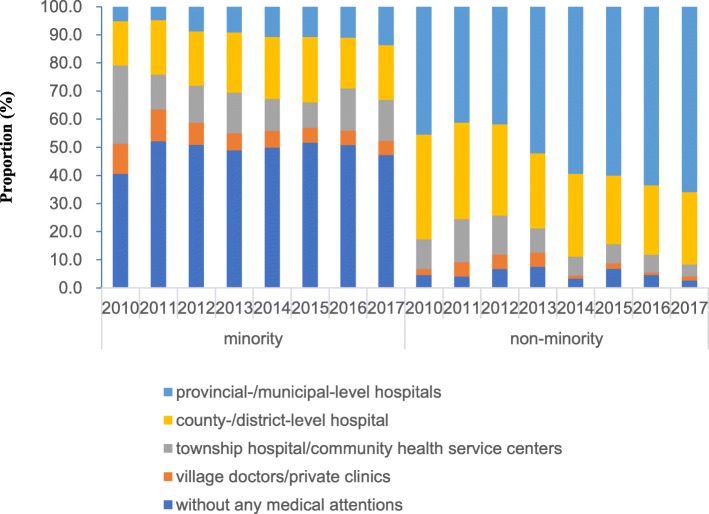
Proportions of medical treatment for children under the age of five years before death in minority and nonminority counties

There was no significant trend over time in the proportions of untreated children before death in the minority and nonminority counties or in the proportion of children treated in county/district hospitals in the minority counties (*p* > 0.05). The proportion of children without any treatment before death in the minority counties was close to 50% in 2017. With regard to the minority counties, the proportions of treatment at village doctors/private clinics and at township hospitals/community health service centers decreased from 10.8 to 5.05% and from 27.8 to 14.7%, respectively (all *p* < 0.001). Although the proportion of children treated in provincial/municipal hospitals has increased (*p* < 0.001), it only increased by 13.6% in the minority counties in 2017 (Fig. 3).

## Discussion

Since 2000, substantial funds have been invested by the Sichuan government to improve maternal and child health in the minority areas [17]. Sichuan has made significant progress in improving the survival rate of children. During the study period, the mortality rate of children under the age of 5 years in Sichuan Province decreased by 51.2% (from 16.9‰ to 8.26‰) [21, 22]. The study showed that PU5MR declined in Sichuan Province from 2010 to 2017, especially in the minority counties. However, the RR of pneumonia mortality of minority to non-minority counties only decreased from a high of a seven-fold to a low of a five-fold difference between 2010 to 2017 and in fact increased over the last 2 years of observation. The minority counties remain at orders of magnitude difference in pneumonia mortality rates, and there was still a large gap between the minority and nonminority counties.

The decline of PU5MR was due to social and economic growth, an increase in health and human resources, the improvement of child nutrition, and an increase access to child healthcare [17, 18]. The medical service capacity, health infrastructure, and availability of medical and health services in the minority regions lagged far behind that of the nonminority regions [23]. In addition, more than half of the health input was borne by the county governments, and it was difficult for the minority counties with lower economic conditions to invest in full and on time [24, 25]. Both the PU5MR and the proportion of pneumonia deaths to total deaths in the minority counties were still higher than those in the nonminority counties. Therefore, it is important to increase investment in the minority regions and promote more balanced development. The government needs to give preference to the minority counties in terms of financial, medical and health equipment, and health and human resources investment.

The current study likewise shows that the proportion of pneumonia deaths to total deaths decreased with time in the minority counties but not in the nonminority counties or the whole province. Children are at a greater risk than adults from the many adverse health effects of air pollution. Their bodies, especially their lungs, are rapidly developing and therefore are more vulnerable to inflammation and other damages caused by pollutants [26]. Due to high humidity and weak wind, the ambient air pollution in the Sichuan basin has become very serious in recent years, especially in the urban areas [27, 28]. More than 90% of the nonminority counties are located in the urban areas. The rise of the proportion of pneumonia deaths to total deaths in the nonminority counties can be partly interpreted by this outcome. The relationship between ambient air pollution and the morbidity and mortality of child pneumonia and the relevant countermeasures should be further studied.

In the minority and nonminority counties, PU5MR decreased within each age group, including the 0–28 days age group, 29 days-11 months age group, and 12–59 months age group. The decline in childhood was the most dramatic among the three age groups. This result may be related to the implementation of some policy measures, including the basic public health services, the major public health services and the introduction of pneumococcal 7-Valent conjugate vaccine (PCV7). Since 2009, the implementation of the basic and major public health services project has increased the percentage of systematic care for children and improved people’s health knowledge [29]. After a series of effective strategies for neonates between 1996 and 2013, such as in-hospital delivery and neonatal family visits reported by He et al. [3], the effect of the follow-up measures may be more obvious in childhood. The dramatic decline in childhood can also be partly explained by a lag phase for the indirect effects of PCV7 observed by Steens A et al. in 2013 [30]. Although the mortality rate decreased, disparities in pneumonia-specific U5MR between the minority and nonminority regions still exist. The pneumonia-specific U5MR in the minority regions was significantly higher than that of the nonminority regions in each age group, and it was highest in the minority counties. There are ethnic differences in pneumonia mortality rates in many developed countries, including Australia [31], South Africa [32] and the United States [33]. Particularly, compared with neonates, the RR of postneonates in the minority counties has increased from 2010 to 2017. The highest mortality rate existed in postneonates of the minority regions. Pneumonia-specific U5MR of postneonates in the minority regions was 179.2 per 100,000, which was still higher than that of 162.8 per 100,000 in 2014–2015 of mainland China, let alone Central and Eastern China [8]. Therefore, more attention needs to be paid to postneonates in the minority counties.

Access to health services in developing countries is affected by many factors, including geographic accessibility, availability, financial accessibility, and acceptability [34]. According to the Sichuan Health and Family Planning Statistical Yearbook of 2016 [22], the accessibility of healthcare sources is worse in the minority areas than in the nonminority areas. First, the minority regions are sparsely populated, and the area of the minority regions accounts for more than half of Sichuan Province, while the healthcare facilities accounts for only 19.3% of the total. Second, the number and educational and professional skill levels of the health service providers in the minority regions are poorer than those the nonminority regions and cannot meet the demands of the people living there. From the perspective of the family and the individual, the low household income and low education level of the minorities are both important factors affecting the accessibility to medical service. The number of children untreated before death in the minority regions accounted for 47.2% of the total deaths in 2017, and no downward trend was found in the present study. Meanwhile, the proportion of children treated in the provincial/municipal level hospitals before death was still relatively low in the minority regions.

Although Sichuan achieved the Millennium Development Goal (MDG4) in 2006, the proportion of pneumonia-specific deaths to total under-five-years-of-age deaths was 15.5% in 2017, with a particularly high proportion in the minority regions (28.9%). The Global Goals For Sustainable Development clearly state the goal of eliminating avoidable deaths of newborns and children under 5 years of age by 2030 [35]. It has been shown that a comprehensive set of interventions can effectively prevent and reduce childhood pneumonia [36]. First, exclusive breastfeeding for the first 6 months, appropriate supplementary feeding and supplementation of vitamin A were all recognized as helping to decrease the incidence and reduce the severity of pneumonia [36]. Therefore, a series of interventions combining individual level and group level health promotion should be implemented (e.g., improving the mother’s education degree, enhancing health education, implementing a nutrition enhancement program). Second, vaccines against *Haemophilus influenzae* type b (Hib) and *Streptococcus pneumonia*(*S. pneumoniae*) should be included in the routine childhood immunization program. As the two most frequent childhood pneumonia bacteria, vaccines against Hib and *S. pneumoniae* could decrease the incidence by 22–34% for Hib and 23–35% for *S. pneumoniae* [37]. Third, the fairness and availability within Sichuan Province, especially in the minority counties, should be improved as soon as possible, and a convenient medical network according to the regional characteristics of minority regions needs to be studied and established to improve the treatment of children who are ill from pneumonia. Last, ambient air pollution control should be taken into account in the prevention of childhood pneumonia.

There are some limitations to this study. Since the data were collected from DRSMCH in Sichuan Province and based on the three-level administration network (urban, community-district-city; rural, village-township-county), it is difficult to obtain an accurate diagnosis of pneumonia in children. However, we have taken a series of measures to ensure the accuracy of diagnosis and the quality of the data. First, ICD codes were used for reporting the causes of deaths, allowing the staff to obtain more information about child deaths [18]. Second, the staff working in the reporting system were trained yearly for death classification and ICD coding. Third, the provincial experts in epidemiology and pediatrics conducted quarterly examinations and confirmations of the causes of death. Another limitation to this study is that demographic data were not collected, such as family income per capita, parents’ educational level, and distance from a patient’s home to the medical institutions, which made it impossible to analyze in depth the causes of the PU5MR.

## Conclusions

The PU5MRs substantially declined in both the minority and nonminority counties of Sichuan Province from 2010 to 2017. Although the disparity in PU5MRs between the minority and nonminority counties decreased over time, it still existed. The pneumonia-specific mortality rate of postneonates was the highest. Priority should be given to strategies for preventing and controlling child pneumonia, especially for postneonates, in the minority counties. It is also worth noting that the proportion of pneumonia deaths to total deaths in the nonminority counties increased.

## Data Availability

The datasets analysed during the current study are available from the corresponding author on reasonable request.

## Abbreviations

CMH: Cochran-Mantel-Haensel
DRSMCH: Direct Report System of Maternal and Child Health
Hib: *Haemophilus influenzae* type b
MDG4: Millennium Development Goal
PCV7: pneumococcal 7-Valent conjugate vaccine
PU5MR: Pneumonia-specific mortality rate of children under the age of five years
S.pneumoniae: Streptococcus pneumonia
